# Predictive value of calf circumference, prealbumin, and serum calcium for 28-day mortality in male sepsis patients with nutritional risk: a retrospective study

**DOI:** 10.7717/peerj.21316

**Published:** 2026-05-18

**Authors:** Haodi Luan, Qianqian Liu, Hua Fan, Yahui Guo, Chenxi Cao, Jing Zhao, Jing Lin

**Affiliations:** 1 Clinical Nutrition Department, Critical Care Medicine Department, The Affiliated Hospital of Inner Mongolia Medical University, Hohhot, China; 2 Department of Gastroenterology, The Affiliated Hospital of Inner Mongolia Medical University, Hohhot, China

**Keywords:** Sepsis, Nutritional risk, NRS-2002, Prognostic assessment

## Abstract

**Background:**

Sepsis is a life-threatening organ-dysfunction syndrome driven by infection. A substantial proportion of septic patients present with nutritional risk, a combination that worsens prognosis. Easily accessible early prognostic tools for this high-risk subset are lacking.

**Objective:**

To identify early risk factors for 28-day clinical death in sepsis patients with nutritional risk.

**Methods:**

A retrospective analysis of sepsis patients in the intensive care unit (ICU) at the Affiliated Hospital of Inner Mongolia Medical University (Jan 2022 to Jan 2025). Patients were categorized by 28-day outcome. Univariate and multivariate logistic regression identified risk factors; receiver operating characteristic (ROC) curves evaluated predictive performance.

**Results:**

Among 231 patients, 154 were male (mortality 13.0%) and 77 female (mortality 11.7%). In males, multivariate analysis identified calf circumference, serum prealbumin (PA), and serum calcium (Ca^2+^) as independent risk factors. ROC analysis showed areas under the curve: calf circumference (AUC: 0.837), PA (AUC: 0.822), and Ca^2+^ (AUC: 0.724). Subgroup analysis using cutoffs (calf circumference ≤ 30 cm, PA ≤ 10.3 mg/dL, Ca^2+^ ≤ 1.94 mmol/L) showed associations with higher sequential organ failure assessment (SOFA) score and acute physiology and chronic health evaluation II (APACHE II) score, and 28-day mortality (all *p* < 0.05). For female patients, only univariate comparisons of baseline characteristics were performed, and significant between-group differences were identified (upper arm circumference, calf circumference, abdominal circumference, APACHE II score, SOFA score, red blood cell count, hemoglobin, prealbumin, prothrombin time, international normalized ratio, serum sodium), while multivariable regression was not conducted due to the small number of outcome events.

**Conclusions:**

Calf circumference, PA, and Ca^2+^ are early independent risk factors for 28-day mortality in male sepsis patients with nutritional risk. Values below the specified cutoffs should prompt consideration of earlier, more aggressive interventions. These single-center findings require validation in larger, multi-center prospective studies.

## Introduction

Sepsis is a common critical illness in intensive care unit (ICU), characterized by systemic inflammatory response, immune dysfunction, microcirculatory issues, and multi-organ failure. Population-based studies applying the sepsis-3 definition document an incidence of 747 cases per 100,000 person-years and in-hospital mortality of 15.5% at 30 days and 20.1% at 90 days (Rudd et al., 2020), rising to 34% during COVID-19 surges (Rudd et al., 2020), while a 2023 Chinese cohort reported ICU mortality of 32% and 90-day mortality of 35.5% (La Via et al., 2024). These factors lead to high mortality rates and poor prognoses for patients (Rudd et al., 2020).

The baseline prevalence of nutritional risk among ICU patients with sepsis worldwide ranges from 38% to 78%, a span driven by regional and case-mix heterogeneity (Stello et al., 2023). Recent international literature (2022–2024) clarifies this dispersion: European cohorts screened with nutritional risk screening 2002 (NRS 2002) ≥ 3 report 46% (Fleischmann-Struzek et al., 2020), East-Asian centres using the same NRS 2002 ≥ 3 threshold record 62% (Liu et al., 2022) and Korean ICUs applying modified nutritional risk in the critically ill (mNUTRIC) ≥ 5 report 52% high nutritional risk (Park et al., 2023), whereas Brazilian ICUs applying mNUTRIC ≥ 5 yield 73% (Yildirim, Yildirim & Deniz, 2024). Sepsis-induced hypermetabolism and hypercatabolism result in increased energy expenditure and accelerated protein breakdown in affected patients, which subsequently exacerbates the risk of malnutrition (Alverdy, 2018). Malnutrition weakens immune function, reduces resistance to infections, and impairs immune regulation in sepsis patients. It worsens organ dysfunction, prolongs ICU hospitalization, and increases morbidity and mortality rates (Hung et al., 2019).

Early identification and intervention for potential risk factors in patients with sepsis, particularly those with nutritional risk, is crucial for enhancing their prognosis. Recent studies have shown that the prognostic nutritional index (PNI) and the nutritional risk in the critically ill (NUTRIC) score are relevant nutritional indicators for predicting outcomes in sepsis patients. PNI, calculated from serum albumin (ALB) and lymphocyte counts (LYM), is significantly associated with increased 60-day mortality (Pan et al., 2025). The NUTRIC score comprises six components: age, acute physiology and chronic health evaluation II (APACHE II) score, sequential organ failure assessment (SOFA) score, number of comorbidities, days from hospital admission to ICU admission, and interleukin-6 (IL-6) level; a high NUTRIC score (≥5) is closely linked to prolonged ICU stay, greater need for mechanical ventilation, and higher mortality (Wełna et al., 2023). To date, prognostic models have lacked anthropometric parameters, but emerging research has begun to explore the use of bedside physical assessment as a convenient and noninvasive tool in the intensive care setting. Recent studies have begun exploring physical assessment measures in ICU settings as accessible and noninvasive tools for evaluation. Building on this line of inquiry, joint refinement of the NUTRIC score has shown that triceps skinfold thickness raises the 28-day mortality area under the curve (AUC) to 0.92 (Prakeerth & Krishna, 2024), while calf circumference reliably estimates height for setting mechanical ventilation (L’Her, Martin-Babau & Lellouche, 2016). The approach is easy to perform, carries minimal cross-contamination risk, and offers an accessible new pathway for nutritional risk screening and individualized therapy in the ICU.

This study aims to investigate the predictive value of nutritional laboratory indicators and physical measurements regarding the 28-day outcomes of ICU patients with sepsis and nutritional risk, providing a foundation for early clinical intervention.

## Methods

### Study design and participants

From May 2025 to July 2025, we accessed the Medu Cloud database of the Affiliated Hospital of Inner Mongolia Medical University to extract relevant data indicators covering the period from January 2022 to January 2025. The study was approved by the ethics committee of the Affiliated Hospital of Inner Mongolia Medical University (approval number: KY2025177) with a waiver of individual informed consent, and registered on ClinicalTrials.gov (registration number: NCT07278167). The study data were extracted from the hospital’s pre-established patient database, for which written informed consent had been obtained from all patients during database construction, and all data were anonymized and de-identified in compliance with ethical regulations. If the patients had been admitted to ICU several times, only the clinical data of the first ICU admission were collected. Inclusion criteria: (1) met the diagnostic criteria for sepsis 3.0 (Singer et al., 2016); (2) met the diagnostic criteria for nutritional risk (NRS 2002 score ≥ 3) (Kondrup et al., 2003); (3) ICU stay ≥ 2 d; (4) age ≥ 18. Exclusion criteria: (1) edema; (2) tumor patients; (3) pregnancy; (4) lack of complete laboratory results. The included patients were categorized into survival and death groups based on their clinical outcomes within 28 days of ICU admission.

### Anthropometric measurements

The study employed a rigorous methodological design. All anthropometric data for enrolled patients were collected within 1 h of admission to the ICU by dietitians from the hospital’s Clinical Nutrition Department. The measurement procedures strictly adhered to the principles and standards outlined in ISO 7250-1:2017 (International Organization for Standardization, 2017).

The data collection sequence was as follows: height, weight, upper arm circumference, abdominal circumference, calf circumference, and triceps skinfold thickness.

#### Height

For ICU bedridden patients unable to stand, height was measured using the recumbent length method. The tool used was a medical-grade non-elastic fiberglass tape (Brand: Seca; Model: Seca 200 Distance Measuring Tape). The patient lay flat without a pillow, with lower limbs naturally extended and together, toes pointing upward. One person fixed the “0” point of the 2-meter tape at the vertex of the head and extended it along the midsagittal plane to the lowest point of the heel. Another person ensured the knees were gently pressed straight and the tape was not curved. The reading was taken at the heel and recorded to 0.1 cm. If the difference between two measurements was ≤ 0.5 cm, the mean was taken; otherwise, a third measurement was taken. The entire procedure could be completed within 1 min.

#### Weight

For ICU bedridden patients, weight was measured using the bed scale method, as the hospital beds were equipped with integrated weighing functionality (Brand: HECAL; Model: Multifunctional Electric Bed LS-EA5028B). Prior to patient transfer, the bed sensor was calibrated using a 50 kg standard weight. If the error was >±0.2 kg, the sensor was recalibrated. After the patient was transferred to the bed, they lay flat without a pillow, with limbs centered. Infusion pumps were paused, and the bed frame was immobilized. The “weight mode” was activated, and the reading was locked once the value stabilized (fluctuation ≤ 0.1 kg). Weight was measured three times consecutively. If the range (difference between the highest and lowest values) among the three measurements was ≤ 0.2 kg, the mean was calculated. If the range was >0.2 kg, the bed was re-zeroed, and three more measurements were taken. The final mean value was recorded as the bed weight.

#### Upper arm circumference

The patient was supine, with the right arm hanging naturally at the side, palm facing forward. A non-elastic fiberglass tape (Brand: Seca; Model: Seca 200) was placed horizontally around the midpoint of the line connecting the acromion and the olecranon. The tape was snug against the skin without compressing underlying tissue. The reading was recorded to 0.1 cm. It was measured twice consecutively; if the difference was ≤ 0.2 cm, the mean was taken; otherwise, a third measurement was taken to minimize error.

#### Abdominal circumference

The patient lay supine without a pillow, with lower limbs naturally extended and relaxed. The tape (Brand: Seca; Model: Seca 200) was placed horizontally around the abdomen at the level of the midpoint between the lower rib margin and the iliac crest along the midaxillary line. The tape was snug against the skin without compression. The reading was taken at the end of a normal expiration and recorded to 0.1 cm. It was measured twice consecutively; if the difference was ≤ 0.5 cm, the mean was taken; otherwise, a third measurement was taken to minimize error.

#### Calf circumference

The patient lay supine with the knee flexed at 90° and the foot flat on the bed surface. A non-elastic tape (Brand: Seca; Model: Seca 200) was placed horizontally around the calf 10 cm below the lower border of the patella. The tape was snug against the skin without compression. The reading was recorded to 0.1 cm. It was measured twice consecutively; if the difference was ≤ 0.2 cm, the mean was taken; otherwise, a third measurement was taken.

#### Triceps skinfold thickness

The patient was supine or seated, with the right arm hanging naturally. At the posterior aspect of the midpoint of the line connecting the acromion and the olecranon, the skin and subcutaneous tissue were pinched vertically between the thumb and forefinger, aligning the skinfold parallel to the long axis of the arm. A calibrated skinfold caliper (Brand: Harpenden; Model: Harpenden Skinfold Caliper) with a constant pressure of 10 g/mm^2^ was applied perpendicularly, one cm from the pinching fingers. The caliper jaws were released for 2–3 s before the reading was taken to 0.1 mm. It was measured twice consecutively at the same site; if the difference was ≤ 0.2 mm, the mean was taken; otherwise, a third measurement was taken.

### Outcome measures

The following baseline patient data was collected: age, sex, vital signs, smoking and alcohol histories, history of underlying diseases (hypertension, diabetes, heart failure, respiratory, gastrointestinal, genitourinary, endocrine, neurologic, hematologic, musculoskeletal, and rheumatic/immune disorders); nutritional parameters (height, weight, upper arm circumference, calf circumference, abdominal circumference, and triceps skinfold thickness); Acute physiology and chronic health evaluation II (APACHE II); APACHE II score is a classic scoring system for assessing the severity of illness and predicting mortality risk in adult patients in the intensive care unit. It calculates a total score (ranging from 0 to 71) based on the worst values of 12 acute physiological parameters (such as temperature, blood pressure, heart rate, respiratory rate, arterial oxygenation, serum electrolytes, *etc.*) within the first 24 h of ICU admission, along with the patient’s age and pre-existing chronic health conditions. A higher score indicates more severe illness and a greater risk of mortality) (Knaus et al., 1985), sequential organ failure assessment (SOFA); SOFA score is a tool for dynamically assessing and quantifying the degree of multiple organ system failure in patients. It focuses on six organ systems: respiratory system (oxygenation index), coagulation system (platelet count), liver (bilirubin), cardiovascular system (blood pressure or use of vasoactive drugs), central nervous system (Glasgow coma scale score), and kidneys (creatinine or urine output). The total score ranges from 0 to 24, and an increase or persistently high score indicates a poor prognosis and higher mortality) (Lambden et al., 2019), and Nutritional risk screening 2002 (NRS 2002); The scoring system (ranging from 0 to 7) integrates the patient’s degree of impaired nutritional status (such as weight loss and reduced food intake) with the severity of the disease. A total score of ≥ 3 indicates the presence of nutritional risk) (Kondrup et al., 2003); duration of mechanical ventilation; ICU length of stay; mortality; and laboratory values drawn from the first set obtained within 24 h of ICU admission.

### Statistical analyses

Statistical analyses were carried out with SPSS 22.0 (IBM Corp, Armonk, NY, USA). Normality and homogeneity of variance were tested for all variables. Continuous variables following normal distribution are expressed as mean ± SD, and non-normally distributed continuous variables are expressed as median with interquartile range (median (P25, P75)). Between-group comparisons for normally distributed continuous data were performed using the independent-samples *t*-test, while non-normally distributed continuous data were analyzed by Mann–Whitney U tests, and categorical variables are expressed as n (%) and were compared with the *χ*^2^ test. Patient clinical outcome served as the dependent variable. Variables that were statistically significant in the univariate analysis were introduced into a multivariate binary logistic regression model to determine independent predictors. Significant predictors identified by the regression were further subjected to receiver operating characteristic (ROC) curve analysis, where the AUC quantified prognostic discrimination for severe cases, to evaluate their prognostic performance in severe cases and to determine optimal cut-off values *via* the Youden index. Owing to the inclusion of anthropometric variables in the baseline dataset, all analyses were conducted stratified by sex to preclude sex-specific confounding. A two-sided *p* < 0.05 was considered statistically significant. All graphs were generated with Origin 2021.

## Results

### Baseline characteristics

Among 368 eligible patients, 137 were excluded because of edema (*n* = 50), malignancy (*n* = 38), pregnancy (*n* = 11), or incomplete laboratory data (*n* = 38), leaving 231 patients for final analysis.

#### Male

Non-survivors were significantly older (median 79.0 *vs* 68.5 years, *p* < 0.01) and presented with greater illness severity, as evidenced by higher APACHE II and SOFA score (both *p* < 0.01). Calf circumference, a marker of nutritional status, was significantly reduced (*p* < 0.01). Laboratory analyses revealed statistically significant alterations, including decreased prealbumin (PA) (*p* < 0.01), elevated C-reactive protein (CRP) (*p* = 0.02), prolonged activated partial thromboplastin time (APTT) (*p* = 0.043), and lower serum calcium (Ca^2+^) (*p* = 0.012). These physiological derangements were associated with significantly longer durations of mechanical ventilation (*p* < 0.01) and extended ICU length of stay (*p* = 0.023).

#### Female

Non-survivors had a median age of 74.0 years, comparable to that of survivors (71.5 years; *p* > 0.05), but exhibited significantly higher APACHE II and SOFA score (*p* < 0.01). Nutritional parameters were consistently lower, with significant reductions in upper arm circumference, calf circumference, and abdominal circumference (*p* ≤ 0.048). Laboratory analyses revealed statistically significant decreases in hemoglobin (Hb) (*p* < 0.01), red blood cell count (RBC) (*p* = 0.006), and PA (*p* < 0.01), along with elevated international normalized ratio (INR) (*p* = 0.025), prolonged prothrombin time (PT) (*p* = 0.021), and increased serum sodium (Na^+^) (*p* = 0.044). No significant differences were observed in duration of mechanical ventilation or ICU length of stay. These results are summarized in Table 1.

**Table 1:** Baseline characteristics of the study participants.

Characteristic	Male (*N* = 154)			Female (*N* = 77)		
	Survive (*n* = 132)	Death (*n* = 22)	*p*	Survive (*n* = 70)	Death (*n* = 7)	*p*
Demographics						
Age, years, median (IQR)	68.5 (57.0, 75.0)	79.0 (71.0, 84.3)	<0.001^**^	71.5 (59.8, 80.3)	74.0 (41.0, 89.0)	0.756
Comorbidities, n (%)						
Hypertension	41 (46.1)	6 (54.6)	0.595	39 (55.7)	6 (85.7)	0.257
Diabetes	25 (28.1)	4 (36.4)	0.827	18 (25.7)	2 (28.6)	0.774
Heart failure	33 (37.1)	7 (63.6)	0.171	22 (31.4)	2 (28.6)	0.785
Pulmonary disease	34 (38.2)	5 (45.5)	0.891	19 (27.1)	2 (28.6)	0.716
Gastrointestinal disease	29 (32.6)	2 (18.2)	0.529	29 (41.4)	2 (28.6)	0.797
Renal disease	24 (27.0)	2 (18.2)	0.793	17 (24.3)	1 (14.3)	0.898
Endocrine disease	6 (6.7)	0 (0.0)	1.000	9 (12.9)	0 (0.0)	0.590
Neurological disease	31 (34.8)	8 (72.7)	0.035^*^	22 (31.4)	3 (42.9)	0.847
Hematologic disease	3 (3.4)	0 (0.0)	1.000	2 (2.9)	0 (0.0)	1.000
Musculoskeletal disease	2 (2.3)	0 (0.0)	1.000	4 (5.7)	0 (0.0)	1.000
Autoimmune disease	3 (3.4)	0 (0.0)	1.000	6 (8.6)	1 (14.3)	0.502
Personal history						
Smoking	29 (32.6)	6 (54.6)	0.269	0	0	–
Alcohol abuse	42 (47.2)	7 (63.6)	0.303	11 (15.7)	0 (0.0)	0.571
Nutritional dimension parameters						
Height, cm, median (IQR)	171.0 (168.0, 175.0)	172.0 (170.0, 175.8)	0.201	160.0 (157.0, 162.0)	162.0 (157.0, 165.0)	0.369
Weight, kg, median (IQR)	68.0 (55.8, 75.0)	67.5 (55.0, 70.6)	0.555	58.5 ± 13.7	54.9 ± 7.6	0.492
BMI, median (IQR)	22.4 (20.0, 25.1)	22.1 (18.7, 24.2)	0.307	23.0 ± 5.4	21.1 ± 2.6	0.359
Upper arm circumference^a^, cm, median (IQR)	27.0 (24.9, 29.6)	26.3 (23.4, 27.1)	0.059	27.0 ± 4.6	22.7 ± 3.7	0.017^*^
Calf circumference^a^, cm, median (IQR)	31.0 (28.4, 34.0)	27.3 (25.5, 28.1)	<0.001^**^	30.7 ± 4.7	23.9 ± 4.4	<0.001^**^
Abdominal circumference^a^, cm, median (IQR)	84.0 (77.4, 91.0)	82.8 (72.8, 89.0)	0.397	85.0 (79.2, 91.5)	79.0 (68.0, 84.6)	0.048^*^
Triceps skinfold thickness^a^, mm, mean ± SD	15.0 ± 6.3	14.3 ± 5.2	0.711	16.5 ± 6.5	15.6 ± 7.5	0.731
Disease severity scores						
APACHE II score, median (IQR)	18.0 (15.0, 21.0)	23.5 (20.0, 25.0)	<0.001^**^	18.0 (15.0, 20.0)	22.0 (22.0, 25.0)	0.001^**^
SOFA score, median (IQR)	7.0 (5.0, 8.0)	8.0 (7.8, 10.0)	<0.001^**^	6.0 (5.0, 8.0)	8.0 (7.0, 12.0)	0.007^**^
NRS 2002 score, median (IQR)	5.0 (4.0, 6.0)	6.0 (4.8, 6.0)	0.008^**^	5.0 (4.0, 6.0)	5.0 (4.0, 5.0)	0.847
Laboratory indices						
WBC, ×10^9^ /L, median (IQR)	11.1 (7.7, 15.5)	9.5 (5.1, 14.4)	0.174	10.2 (7.8, 13.8)	14.5 (9.9, 17.3)	0.156
RBC, ×10^12^ /L, mean ± SD	4.0 ± 1.1	3.6 ± 0.6	0.310	3.8 ± 0.9	2.9 ± 0.4	0.006^**^
HGB, g/L, mean ± SD	123.0 ± 32.4	118.6 ± 20.8	0.658	114.7 ± 23.6	85.9 ± 8.7	0.002^**^
PLT, ×10^9^ /L, median (IQR)	177.5 (127.0, 239.0)	143.0 (101.5, 194.3)	0.061	182.5 (108.5, 273.5)	137.0 (90.0, 183.0)	0.199
MPV, fL, median (IQR)	10.4 (9.4, 11.1)	10.8 (9.8, 11.6)	0.130	10.0 ± 1.4	10.4 ± 1.4	0.529
NEU, ×10^9^ /L, median (IQR)	9.4 (6.6, 13.2)	7.8 (5.0, 11.3)	0.231	8.6 (6.5, 12.6)	11.8 (9.6, 15.6)	0.128
CRP, mg/L, median (IQR)	73.6 (31.5, 127.0)	104.8 (64.1, 184.0)	0.020^*^	52.9 (18.5, 123.6)	101.1 (67.2, 184.6)	0.060
PCT, ng/mL, median (IQR)	0.5 (0.2, 4.0)	1.1 (0.3, 3.0)	0.448	0.3 (0.1, 1.4)	1.6 (0.3, 6.1)	0.063
ALT, IU/L, median (IQR)	28.1 (16.0, 54.8)	18.0 (10.7, 62.5)	0.114	20.7 (12.2, 32.0)	10.0 (7.6, 48.0)	0.130
AST, IU/L, median (IQR)	32.0 (22.0, 67.9)	44.0 (21.5, 76.3)	0.397	27.0 (19.0, 49.5)	24.0 (12.0, 55.0)	0.395
TBIL, umol/L, median (IQR)	16.1 (11.4, 30.1)	14.0 (10.9, 16.4)	0.071	14.8 (9.4, 22.4)	10.6 (7.9, 33.6)	0.901
GGT, IU/L, median (IQR)	46.5 (22.4, 99.1)	41.3 (30.4, 78.5)	0.724	27.1 (16.9, 55.1)	11.0 (9.0, 57.9)	0.139
ALP, IU/L, median (IQR)	70.0 (51.0, 101.0)	72.5 (45.5, 102.3)	0.693	62.5 (49.8, 87.3)	50.0 (37.0, 58.0)	0.113
ALB, g/L, mean ± SD	31.4 ± 6.4	31.8 ± 4.8	0.859	32.0 ± 5.0	33.3 ± 4.6	0.524
PA, mg/dL, median (IQR)	13.5 (8.6, 20.6)	5.7 (3.3, 9.6)	<0.001^**^	13.6 (7.4, 17.5)	6.4 (4.1, 8.7)	0.003^**^
GLB, g/L, median (IQR)	23.8 (19.6, 28.1)	25.5 (21.7, 31.4)	0.063	25.1 (20.6, 28.9)	21.7 (16.2, 30.6)	0.506
TC, mmol/L, median (IQR)	3.2 ± 1.2	3.3 ± 0.5	0.645	3.5 (2.8, 4.3)	3.9 (3.2, 4.9)	0.385
TG, mmol/L, median (IQR)	1.2 (0.7, 1.7)	1.1 (0.8, 1.5)	0.843	1.4 (0.9, 2.1)	1.8 (0.7, 2.7)	0.366
GLU, mmol/L, median (IQR)	7.5 (5.7, 10.0)	7.8 (5.0, 10.2)	0.875	7.0 (5.0, 8.5)	7.8 (7.2, 9.7)	0.250
CREA, umol/L, median (IQR)	78.5 (60.3, 114.0)	83.0 (57.0, 167.3)	0.646	53.0 (42.0, 77.0)	66.0 (63.0, 117.0)	0.069
UA, umol/L, median (IQR)	303.5 (192.8, 446.0)	274.5 (191.0, 547.8)	0.774	239.0 (151.0, 345.8)	283.0 (185.0, 389.0)	0.607
BUN, mmol/L, median (IQR)	8.9 (6.7, 14.3)	12.0 (6.5, 18.4)	0.357	6.4 (4.5, 9.1)	7.8 (4.2, 16.4)	0.595
PT, second, median (IQR)	13.4 (12.0, 14.9)	13.6 (12.5, 15.4)	0.351	12.6 (11.5, 14.1)	13.9 (13.3, 18.3)	0.021^*^
APTT, second, median (IQR)	29.1 (26.9, 32.5)	32.3 (28.2, 35.5)	0.043^*^	28.3 (25.8, 32.7)	29.2 (27.6, 47.7)	0.154
INR, median (IQR)	1.2 (1.1, 1.3)	1.2 (1.1, 1.4)	0.428	1.1 (1.0, 1.3)	1.2 (1.2, 1.6)	0.025^*^
FIB, g/L, median (IQR)	4.4 (2.7, 5.8)	3.7 (1.9, 5.3)	0.219	4.3 ± 1.9	3.1 ± 1.7	0.106
Na^+^, mmol/L, median (IQR)	139.0 (136.0, 143.0)	137.0 (134.0, 139.7)	0.037^*^	138.9 (136.0, 141.0)	142.0 (139.0, 147.0)	0.044^*^
Ca^2+^, mmol/L, median (IQR)	2.0 (1.9, 2.1)	1.8 (1.7, 1.9)	0.012^*^	2.0 (1.9, 2.1)	2.1 (1.9, 2.1)	0.500
K^+^, mmol/L, mean ± SD	3.9 (3.4, 4.4)	4.1 (3.4, 4.7)	0.544	3.6 ± 0.6	3.7 ± 0.6	0.781
Ventilator days, median (IQR)	3.8 (1.6, 7.0)	6.2 (2.0, 15.4)	0.048^*^	2.7 (0.2, 6.1)	3.9 (2.8, 9.3)	0.298
ICU hospitalization days, median (IQR)	7.7 (4.6, 15.0)	15.5 (7.2, 20.2)	0.023^*^	7.9 (4.9, 12.2)	7.0 (3.6, 15.1)	0.723

**Notes.**

* *p* < 0.05.

** *p* < 0.01.

Data are number (%), mean ± standard deviation or median [interquartile range].

Abbreviations APACHE II acute physiology and chronic health evaluation II SOFA sequential organ failure assessment NRS 2002 nutritional risk screening 2002 BMI body mass index IQR interquartile range SD standard deviation WBC white blood cell count RBC red blood cell count HGB hemoglobin PLT platelet count MPV mean platelet volume NEU neutrophil count CRP C-reactive protein PCT procalcitonin ALT alanine aminotransferase AST aspartate aminotransferase TBil total bilirubin GGT *γ*-glutamyl transferase ALP alkaline phosphatase ALB albumin PA prealbumin GLB globulin TC total cholesterol TG triglycerides GLU glucose CREA creatinine UA uric acid BUN blood urea nitrogen PT prothrombin time APTT activated partial thromboplastin time INR international normalized ratio FIB fibrinogen Na^+^ serum sodium Ca^2^^+^ serum calcium K^+^ serum potassium

### Logistic regression analysis in male patients

Owing to the small number of female patients with death events and the potential for bias, the analysis was only performed in the male subgroup. Multivariate logistic regression analysis using indicators with statistically significant differences in univariate analysis, including calf circumference, CRP, PA, APTT, Na^+^, and Ca^2+^ as the independent variables, and clinical death as the dependent variable, showed that lower calf circumference, PA and Ca^2+^ were the independent risk factors (all *p* < 0.05), as detailed in Table 2. Given the small sample size of non-survivors, which may introduce bias into the analysis, we conducted a collinearity assessment for all included variables; the variance inflation factor (VIF) for each was <5, as presented in Table 3.

**Table 2:** Collinearity analysis.

Characteristic	Regression coefficient	Standard error	OR	OR (95% CI)	*p*
Calf circumference (cm)	−0.555	0.162	0.574	0.418∼0.788	0.001^**^
CRP (mg/L)	0.004	0.006	1.004	0.992∼1.016	0.531
PA (mg/dL)	−0.277	0.095	0.758	0.630∼0.912	0.003^**^
APTT (second)	−0.026	0.044	0.975	0.895∼1.062	0.557
Na^+^ (mmol/L)	−0.055	0.052	0.947	0.854∼1.049	0.296
Ca^2+^ (mmol/L)	−3.634	1.441	0.026	0.002∼0.445	0.012^*^

**Notes.**

* *p* < 0.05.

** *p* < 0.01.

Abbreviations OR odds ratio CI confidence interval CRP C-reactive protein PA prealbumin APTT activated partial thromboplastin time Na^+^ serum sodium Ca^2+^ serum calcium

**Table 3:** Summary of binary logit regression analysis results.

Characteristic	VIF	Tolerance
CRP (mg/L)	1.071	0.934
PA (mg/dL)	1.112	0.899
APTT (second)	1.090	0.917
Na^+^ (mmol/L)	1.075	0.930
Ca^2+^ (mmol/L)	1.077	0.929
Calf circumference (cm)	1.085	0.921

**Notes.**

Abbreviations VIF variance inflation factor CRP C-reactive protein PA prealbumin APTT activated partial thromboplastin time Na^+^ serum sodium Ca^2^^+^ serum calcium

### ROC curve analysis in male patients

**Figure 1:**
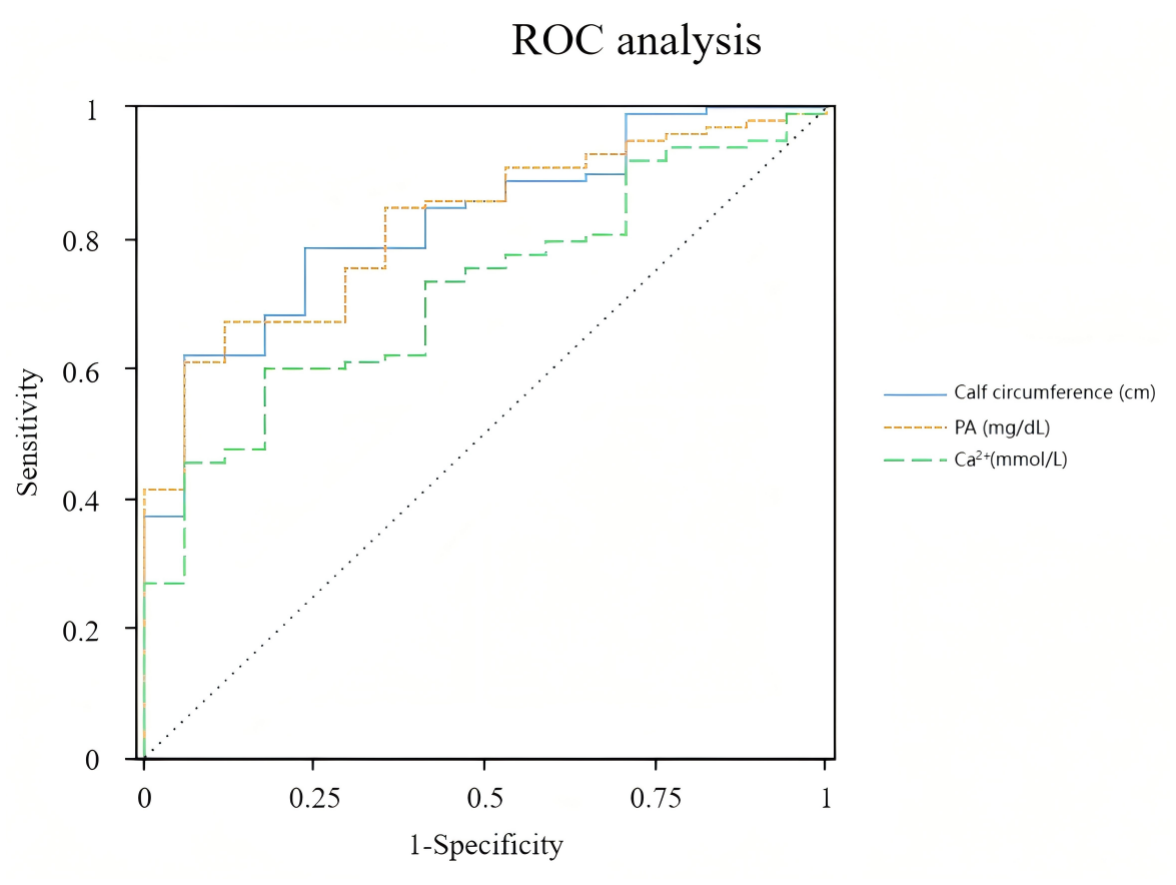
ROC curve analysis in male patients.

The AUC of calf circumference PA and Ca^2+^ for predicting clinical death within 28 days of admission to the ICU in patients with sepsis combined with nutritional risk were 0.837 (95% CI [0.750∼0.925]), 0.822 (95% CI [0.731∼0.912]) and 0.724 (95% CI [0.613∼0.835]), respectively. The best cut-off value for calf circumference was 30 cm, with a sensitivity of 61.9% and a specificity of 94.1% for predicting clinical death within 28 days of ICU admission in patients with sepsis combined with nutritional risk. The best cutoff value for PA was 10.3 mg/dL, with a sensitivity of 67% and a specificity of 88.2% for predicting clinical death within 28 days of ICU admission in patients with sepsis combined with nutritional risk. The best cutoff value for Ca^2+^ was 1.94 mmol/L, with a sensitivity of 59.8% and a specificity of 82.4% for predicting clinical death within 28 days of ICU admission in patients with sepsis combined with nutritional risk, as presented in Fig. 1 and Table 4.

**Table 4:** Results of the ROC analysis for male patients.

Characteristic	AUC	AUC (95% CI)	Cut-off	Sensitivity	Specificity	*p*
Calf circumference (cm)	0.837	0.750 ∼0.925	30	0.619	0.941	<0.001^**^
PA (mg/dL)	0.822	0.731 ∼0.912	10.3	0.67	0.882	<0.001^**^
Ca^2+^ (mmol/L)	0.724	0.613 ∼0.835	1.94	0.598	0.824	0.003^**^

**Notes.**

** *p* < 0.01.

Abbreviations AUC area under the curve CI confidence interval PA prealbumin Ca^2+^ serum calcium

### Subgroup analysis based on the optimal cutoff value for calf circumference, PA and Ca^2+^

Based on the optimal calf circumference cut-off of 30 cm, the 154 septic patients with nutritional risk were divided into two groups. Patients with calf circumference ≤ 30 cm exhibited higher SOFA, APACHE II and NRS 2002 score than those with calf circumference >30 cm (*p* < 0.05). Moreover, the 28-day ICU mortality rate was significantly higher in the calf circumference ≤ 30 cm group than in the circumference > 30 cm group (28.77% *vs.* 1.23%).

Based on the optimal PA cut-off of 10.3 mg/dL, the 154 septic patients with nutritional risk were divided into two groups. Patients with PA ≤ 10.3 mg/dL exhibited higher SOFA, APACHE II and NRS 2002 score than those with PA >10.3 mg/dL (*p* < 0.05). Moreover, the 28-day ICU mortality rate was significantly higher in the PA ≤ 10.3 mg/dL group than in the PA > 10.3 mg/dL group (30.77% *vs.* 2.25%).

Based on the optimal Ca^2+^ cut-off of 1.94 mmol/L, the 154 septic patients with nutritional risk were divided into two groups. Patients with Ca^2+^ ≤ 1.94 mmol/L exhibited higher 28-day ICU mortality rate than patients with Ca^2+^ > 1.94 mmol/L group (20.43% *vs.* 4.92%). These results are presented in Table 5.

**Table 5:** Subgroup analysis based on the cut-off point.

Characteristic	Calf circumference (cm) (*N* = 154)				PA (mg/dL) (*N* = 154)				Ca^2+^ (mmol/L) (*N* = 154)			
	>30 (*n* = 81)	≤30 (*n* = 73)	*Z*/*χ2*	*p*	>10.3 (*n* = 89)	≤10.3 (*n* = 65)	*Z*/*χ2*	*p*	>1.94 (*n* = 61)	≤1.94 (*n* = 93)	*Z*/*χ2*	*p*
Sofa	6.0 (5.0, 8.0)	7.0 (6.0, 8.0)	−2.392	0.017^*^	6.0 (5.0, 8.0)	8.0 (6.0, 8.0)	−3.100	0.002^**^	7.0 (5.0, 8.0)	7.0 (5.0, 8.0)	−0.245	0.807
APACHE II	18.0 (15.0, 21.0)	20.0 (18.0, 23.5)	−2.485	0.013^*^	18.0 (15.0, 20.0)	20.0 (18.0, 25.0)	−3.297	0.001^**^	18.0 (15.5, 22.0)	20.0 (15.0, 22.0)	−0.129	0.897
NRS 2002	5.0 (4.0, 5.0)	5.5 (5.0, 6.0)	−3.286	0.001^**^	5.0 (4.0, 6.0)	5.0 (4.5, 6.0)	−2.131	0.033^*^	5.0 (4.0, 6.0)	5.0 (4.0, 6.0)	−0.263	0.792
Mechanical ventilation duration (d)	3.8 (1.6, 7.6)	4.1 (2.0, 7.0)	−0.621	0.535	3.7 (1.2, 7.0)	4.6 (2.0, 7.7)	−1.131	0.258	4.1 (1.2, 6.8)	4.0 (2.0, 9.3)	−1.100	0.272
ICU duration of stay (d)	8.5 (4.0, 16.8)	10.0 (5.7, 16.7)	−1.044	0.296	7.9 (4.2, 14.1)	10.2 (5.8, 17.5)	−1.769	0.077	9.0 (5.1, 14.5)	8.8 (4.6, 18.0)	−0.497	0.619
ICU 28-day mortality rate	1 (1.2)	21 (28.8)	23.770	<0.001^**^	2 (2.3)	20 (30.8)	24.957	<0.001^**^	3 (4.9)	19 (20.4)	7.239	0.007^**^

**Notes.**

* *p* < 0.05.

** *p* < 0.01.

Abbreviations SOFA sequential organ failure assessment APACHE II acute physiology and chronic health evaluation II NRS 2002 nutritional risk screening 2002 PA prealbumin Ca^2+^ serum calcium ICU intensive care unit Z/*χ*2 Z/Chi-square value

**Figure 2:**
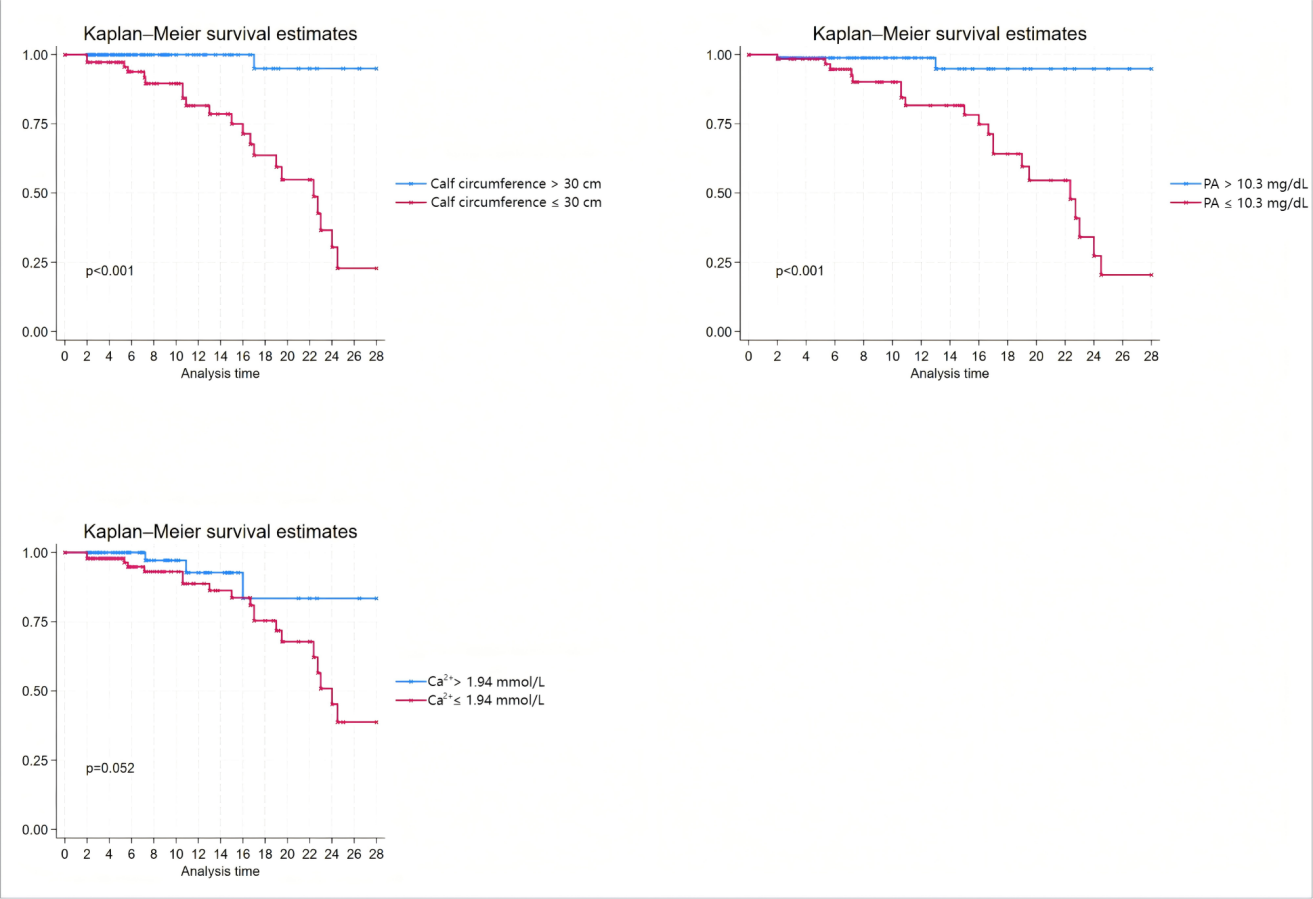
Kaplan–Meier survival analysis.

Based on the respective cutoff values, we performed a survival analysis; calf circumference and PA showed significant associations with survival (*p* < 0.05). For Ca^2+^, the statistical result was *p* = 0.052, yet a discernible survival trend was observed, as presented in Fig. 2.

## Discussion

Early identification of potential risk factors in patients with sepsis combined with nutritional risk is essential to improve prognosis. Related studies have reported that the ICU 28-day mortality rate in patients with sepsis combined with nutritional risk can be 15.8%–33.2% (Hung et al., 2019; Oh & Song, 2023; Safabakhsh et al., 2024). Our observed ICU 28-day mortality rate of 12.6% falls within the broad range reported in current related studies (15%–55%) and is close to the lower end of this interval. For example, it is similar to the overall mortality rate of 15.8% reported in a large real-world study involving 9,763 septic patients conducted in South Korea (Oh & Song, 2023). This discrepancy primarily stems from the heterogeneity of the study populations: many studies reporting higher mortality rates typically focus on specific extremely high-risk subgroups. For instance, a 2024 study reported that in patients with septic shock, the 28-day mortality rate in the nutritional risk subgroup can reach 33.2% (Safabakhsh et al., 2024). In contrast, the baseline characteristics of our patient cohort are likely more comparable to the broader spectrum of septic ICU patients included in the aforementioned large-scale study. Furthermore, the adherence of our medical center to international treatment guidelines and the level of early bundle therapy implementation are important factors contributing to this difference and support the reasonableness of our data. Therefore, our data reflect the prognosis within a specific clinical context and patient spectrum, which is reasonable and consistent with results from studies of similar disease severity in the literature.

Calf circumference is a key indicator of muscle reserve and nutritional status, with significant clinical relevance for assessing malnutrition, sarcopenia, and disease prognosis (Hung et al., 2019). It is easy to measure and non-invasive, directly reflecting changes in lower limb muscle mass (Ceolin et al., 2024). In nutritional assessments, calf circumference is closely linked to sarcopenia and malnutrition outcomes. Muscle loss, indicated by reduced calf circumference, is a hallmark of malnutrition (Landi et al., 2019). For example, calf circumference <34 cm in men or <33 cm in women suggests high sarcopenia risk (Kim, Kim & Kim, 2024). This muscle loss can impair physical strength, endurance, and increase infection risk (Zhang et al., 2021). For critically ill patients, calf circumference serves as a practical proxy for muscle mass and nutritional status when more precise methods (*e.g.*, CT, MRI, body composition analysis) are unavailable.

In clinical practice, measuring body dimensions like abdominal and calf circumferences can be prone to errors (Verweij et al., 2013), due to measurer differences, inconsistent methods, and subject variability. These errors can impact clinical decisions and research reliability, and there is often a lack of standardized training (Verweij et al., 2013). This study used the ISO 7250-1:2017 standard (International Organization for Standardization, 2017) and had registered clinical dietitians perform the measurements to reduce errors. While imaging and body composition analysis can precisely measure muscle and fat, they are costly and labor-intensive (Fuchs et al., 2025; Niu et al., 2025a). Calf circumferences are easier to obtain in clinical settings, but clinicians must interpret these metrics based on standardized procedures and consider how measurement errors affect disease outcomes.

Prealbumin is a low-molecular-weight plasma protein synthesized by hepatocytes with a half-life of about 1.9 days, making it more sensitive than albumin in reflecting nutritional and inflammatory status (Sanguinetti et al., 2022). In sepsis, inflammatory mediators inhibit hepatic protein synthesis, causing a significant drop in prealbumin levels (Shang et al., 2024). This decline indicates hepatic inflammation and is closely linked to nutritional risk. Low prealbumin levels suggest poor recent nutritional intake, weakened immune function, and impaired organ repair, increasing infection risk and worsening prognosis (Nabarrete et al., 2021). Studies show that sepsis patients with low prealbumin at admission have higher mortality rates than those with normal levels, consistent with this study (Liu & Liu, 2023). However, prealbumin levels are influenced by multiple factors, including liver and kidney function, and drug metabolism (Liao et al., 2019). In clinical practice, prealbumin should be combined with other indicators and overall clinical presentation to assess nutritional status and prognosis in sepsis patients.

Calcium is one of the most abundant electrolytes in the body. It is tightly regulated by the parathyroid–bone–kidney axis, and its serum half-life is short (minutes to hours), allowing it to mirror acute metabolic and inflammatory shifts more rapidly than albumin (Cao et al., 2025). In sepsis, inflammatory mediators such as tumor necrosis factor-alpha (TNF-α) and IL-6 blunt parathyroid hormone receptor signaling, increase renal calcium excretion, and down-regulate calcium-channel expression, producing a rapid fall in both total and ionized serum calcium (Gong et al., 2024; Mehdi et al., 2025). This decline signals more than an electrolyte imbalance; it flags a high nutritional risk. Hypocalcemia is frequently accompanied by recent protein–energy underfeeding, vitamin D deficiency, and magnesium depletion, which together impair leukocyte chemotaxis and phagocytosis, retard organ repair, raise the likelihood of secondary infections, and worsen outcome (Kumar & Gopaldas, 2024). Several studies have confirmed that septic patients with hypocalcemia at admission have a significantly higher 28-day mortality than those with normal serum calcium levels (Niu, Bai & Zong, 2025b). However, calcium levels are influenced by multiple factors, including acid–base balance, albumin concentration, renal function, and the metabolism of drugs such as proton-pump inhibitors and diuretics (Ahmed et al., 2025; Salcedo-Betancourt & Moe, 2024).

This study has several limitations due to its retrospective design. First, missing data required the use of NRS 2002 instead of ICU-specific NUTRIC score for assessing nutritional risk, and there was a lack of blood gas analysis. Second, only data from the first 24 h after ICU admission were recorded; monitoring additional data over time could provide more valuable insights. Lastly, the retrospective nature of the study, coupled with an extremely small non-survivor sample, limits its generalizability and may introduce bias into the results.

## Conclusions

This retrospective study, conducted at the Affiliated Hospital of Inner Mongolia Medical University from January 2022 to January 2025, demonstrates that calf circumference, serum prealbumin and serum calcium are early independent risk factors for 28-day clinical mortality in male sepsis patients with nutritional risk admitted to the ICU. For those with calf circumference ≤ 30 cm, serum prealbumin ≤ 10.3 mg/dL, or serum calcium ≤ 1.94 mmol/L, clinicians should anticipate an unfavorable outcome and consider earlier initiation of more aggressive therapeutic or nutritional interventions. Nevertheless, these findings are limited by the small sample size and single-center design, and require validation in larger, multi-center prospective studies.

##  Supplemental Information

10.7717/peerj.21316/supp-1 Supplemental Information 1 Glossary

10.7717/peerj.21316/supp-2 Supplemental Information 2 Raw data

## Additional Information and Declarations

### Data Availability

The following information was supplied regarding data availability: The original data is available in the Supplemental File.
