# Supplementary material for: Predictive value of calf circumference, prealbumin, and serum calcium for 28-day mortality in male sepsis patients with nutritional risk: a retrospective study
Source: PeerJ. 2026 May 18;14:e21316. doi: 10.7717/peerj.21316 (PMC13192455; doi:10.7717/peerj.21316)
Supplement: Supplemental Information 1 [file peerj-14-21316-s001.docx]

## Glossary

| AC | Abdominal Circumference |
| --- | --- |
| AC/CC | Abdominal Circumference/Calf Circumference |
| ALB | Albumin |
| APACHE II | Acute Physiology and Chronic Health Evaluation II |
| APTT | Activated Partial Thromboplastin Time |
| ALT | Alanine Aminotransferase |
| ALP | Alkaline Phosphatase |
| AST | Aspartate Aminotransferase |
| AUC | Area Under the Curve |
| BUN | Blood Urea Nitrogen |
| BMI | Body Mass Index |
| CREA | Creatinine |
| CC | Calf Circumference |
| CI | Confidence Interva |
| CRP | C - Reactive Protein |
| CT | Computed Tomography |
| FIB | Fibrinogen |
| GLB | Globulin |
| GLU | Glucose |
| GGT | Gamma - Glutamyl Transferase |
| HGB | Hemoglobin |
| ICU | Intensive Care Unit |
| IL-6 | Interleukin-6 |
| INR | International Normalized Ratio |
| MPV | Mean Platelet Volume |
| MRI | Magnetic Resonance Imaging |
| NEU | Neutrophil Count |
| NRS 2002 | Nutritional Risk Screening 2002 |
| OR | Odds Ratio |
| PA | Prealbumin |
| PLT | Platelet Count |
| PCT | Procalcitonin |
| PT | Prothrombin Time |
| RBC | Red Blood Cell Count |
| ROC | Receiver operating characteristic |
| SOFA | Sequential Organ Failure Assessment |
| TBIL | Total Bilirubin |
| TC | Total Cholesterol |
| TG | Triglycerides |
| TNF-α | Tumor Necrosis Factor-alpha |
| UA | Uric Acid |
| WBC | White Blood Cell Count |
